# Controlled release of artemisone for the treatment of experimental cerebral malaria

**DOI:** 10.1186/s13071-017-2018-7

**Published:** 2017-03-01

**Authors:** Jacob Golenser, Viola Buchholz, Amir Bagheri, Abed Nasereddin, Ron Dzikowski, Jintao Guo, Nicholas H. Hunt, Sara Eyal, Natalia Vakruk, Andreas Greiner

**Affiliations:** 1 Department of Microbiology and Molecular Genetics, The Kuvin Center for the Study of Infectious and Tropical Diseases, The Hebrew University of Jerusalem (HU)-Hadassah Medical School (HMS), Jerusalem, Israel; 2 Macromolecular Chemistry II, University of Bayreuth, Universitätsstrasse 30, Bayreuth, Germany; 3 Institute of Drug Research, School of Pharmacy, HU-HMS, Jerusalem, Israel; 4 Al-Quds University, Abu Dis, The Palestinian Authority; 5 Department of Pathology and Bosch Institute, The University of Sydney, Sydney, Australia; 6 State Key Laboratory of Respiratory Diseases, Guangzhou Institutes of Biomedicine and Health, Chinese Academy of Sciences, Guangzhou, China

**Keywords:** Cerebral malaria, *Plasmodium falciparum*, *Plasmodium berghei* ANKA, Artemisone, Controlled release

## Abstract

**Background:**

Cerebral malaria (CM) is a leading cause of malarial mortality resulting from infection by *Plasmodium falciparum*. Treatment commonly involves adjunctive care and injections or transfusion of artemisinins. All artemisinins that are in current use are metabolized to dihydroxyartemisinin (DHA), to which there is already some parasite resistance. We used artemisone, a derivative that does not convert to DHA, has improved pharmacokinetics and anti-plasmodial activity and is also anti-inflammatory (an advantage given the immunopathological nature of CM).

**Methods:**

We examined controlled artemisone release from biodegradable polymers in a mouse CM model. This would improve treatment by exposing the parasites for a longer period to a non-toxic drug concentration, high enough to eliminate the pathogen and prevent CM. The preparations were inserted into mice as prophylaxis, early or late treatment in the disease course.

**Results:**

The most efficient formulation was a rigid polymer, containing 80 mg/kg artemisone, which cured all of the mice when used as early treatment and 60% of the mice when used as a very late treatment (at which stage all control mice would die of CM within 24 h). In those mice that were not completely cured, relapse followed a latent period of more than seven days. Prophylactic treatment four days prior to the infection prevented CM. We also measured the amount of artemisone released from the rigid polymers using a bioassay with cultured *P. falciparum*. Significant amounts of artemisone were released throughout at least ten days, in line with the in vivo prophylactic results.

**Conclusions:**

Overall, we demonstrate, as a proof-of-concept, a controlled-sustained release system of artemisone for treatment of CM. Mice were cured or if treated at a very late stage of the disease, depicted a delay of a week before death. This delay would enable a considerable time window for exact diagnosis and appropriate additional treatment. Identical methods could be used for other parasites that are sensitive to artemisinins (e.g. *Toxoplasma gondii* and *Neospora caninum*).

## Background

Malaria kills about 600,000 people annually, harms hundreds of millions and causes tremendous economic losses. Cerebral malaria (CM) is a leading cause of the malarial mortality that may follow infection by *Plasmodium falciparum* [1, 2]. CM has an immunopathological etiology; it is induced by inflammatory responses against plasmodia sequestered in the brain vasculature and at the blood brain barrier [3, 4], and is also associated with deleterious changes in brain metabolism [4–9].

In malaria-endemic countries many *P. falciparum* strains have become resistant to most the conventional antimalarial drugs, making development of alternative drugs a necessity. The most recent successful anti-malarial drugs that have been introduced are artemisinin derivatives. However, these drugs (e.g. artemether and artesunate) are converted in vivo to dihydroartemisinin (DHA), which has a short half-life (less than an hour). In contrast, another artemisinin derivative, artemisone, does not metabolize to DHA, has a longer half-life, increased anti-plasmodial activity, and thermal and metabolic stability [10, 11]. Compared to the other derivatives it displays no neurotoxicity [12] but its embryo-toxicity is in debate [13]. In a preclinical study artemisone was profoundly superior to artesunate in treatment of CM [14].

Artemisinins, including artemisone, possess both anti-plasmodial and anti-inflammatory properties, an advantage in the treatment of CM [14–16]. In a mouse model of CM artemisone could prevent CM and inhibit the development of the parasites. However, repeated injections, at least twice a day over several days, were needed to obtain a significant effect [14]. A similar necessity dictates the mode of treatment of CM in humans [12]. In parallel, patient non-compliance is limiting the use of artemisinins, including artemisone. The problem of repeated injections might partially be solved using artemisinin combination therapies (ACTs) that are more effective than individual drugs: combining a fast-acting artemisinin derivative that rapidly clears a large proportion of the parasites within its short pharmacological half-life, with a much longer half-life partner drug that continues the clearance while the artemisinin concentration falls to sub-therapeutic levels [17]. Obviously, the use of ACTs is applied to prevent the induction of resistance. However, recent incidences of resistance to ACTs have been reported [18]. The phenomenon has necessitated at least a two-fold increase in the artemisinin dose in ACT treatment regimens to prolong exposure of blood-stage parasites to the drug [19, 20]. Artemisone would be advantageous for ACTs because of its improved pharmacokinetics [10, 11]. In the current work, we examined a novel option for improved treatment - the use of sustained release formulations. To explore this in more detail we studied the release of artemisone from solid samples of a composite of biodegradable polyester and artemisone and corresponding aqueous dispersions. We hypothesized that this approach would improve the treatment by exposing the parasite for a longer period to a drug concentration sufficiently high to eliminate the pathogens and prevent CM.

## Methods

### Parasites

*Plasmodium berghei* ANKA (PbA) strain (MRA-311, CDC, Atlanta, GA, USA) was maintained in vivo by serial transfer of parasitized erythrocytes from infected to naive mice. To avoid loss of virulence, infection was renewed every six months by using frozen stabilates.

*Plasmodium falciparum* NF54-luc parasites that stably and constitutively express luciferase were cultivated at 5% hematocrit in RPMI 1640 medium, 0.5% Albumax II (Invitrogen, Carlsbad, California, USA), 0.25% sodium bicarbonate, and 0.1 mg/ml gentamicin. Parasites were incubated at 37 °C in an atmosphere of 5% oxygen, 5% carbon dioxide and 90% nitrogen. Parasites were cultured in media containing 4 nM WR99210 to select for stable luciferase expression. Parasite viability assays were performed either by measuring their luciferase activity (see Bioassay below) or by direct microscopic observation of Giemsa (Sigma-Aldrich, St. Louis, USA) stained blood smears.

### Mice

Male C57BL/6 mice (7–8 weeks old) were purchased from Harlan Laboratories (Rehovot, Israel). The mice had free access to a standard diet and water, and they were maintained on a 12/12-h automatically timed light/dark cycle.

### Induction of CM

The validity of the CM model in mice has been demonstrated previously [21–23]. Mice were infected with 80,000 parasitized erythrocytes (this leads to CM in the majority of mice). Parasitemia was monitored by blood smears prepared from the tail vein, stained with Giemsa and examined by light microscopy. Mice were monitored for clinical signs of neuropathology that appear a few days before death from CM (coat staring, hunching and wobbly gait, about 5–6 days post-infection) [4, 14]. Mice that developed further neurological symptoms such as ataxia, paralysis and coma, drastic weight reduction and depicted parasitemia below or about 20%, were considered to have fatal CM. At this stage death was inevitably expected within 24 h and the mice were euthanized. This assumption was confirmed by brain histological sections [21, 24]. Typically, death of CM would occur at day 8–9 post-infection at parasitemia below 20%. Mice, which did not die of CM, did not show these symptoms and would die of severe anemic malaria (AM) related to high parasitemia, above 20%, one to two weeks later [24]. Mice that were about to die of anemic malaria were also euthanized. Mice that were treated with an anti-malarial and consequently did not die of malaria returned to a normal appearance.

### Treatment

The artemisone was introduced in different concentrations into PCL-b-MPEG dispersions or solid samples of PCL-MPEG. The dispersions were intraperitoneally (IP) injected and the solid polymers were inserted subcutaneously into the abdomen of mice anesthetized by Ketamine/Xylazine injection, on different days before or after inoculation of PbA. Treatment before infection was performed to examine whether the in vivo retaining of artemisone is sufficient to affect the course of infection.

### Polymer preparation

Blockpolymer PCL-MPEG was synthesized, according to a previously published procedure [25]. Blockcopolymers of PCL-MPEG were fabricated by different ratios of PCL: MPEG. To create a homogeneous mixture of PCL-*b*-MPEG and artemisone, different ratios of both compounds were dissolved in small amounts of tetrahydrofuran (THF; p.a. ≥ 99.9%). After all particles were dissolved, the solvent was completely evaporated. Using a heat press at 65 °C the mixture was pressed into a polytetrafluoroethylene matrix (internal size about 0.5 × 10 × 20 mm^3^) and then cooled down to room temperature under a second press at about 20 °C. The polymers were sterilized by brief (5 s) washing in 70% ethanol and exposure to UV for 45 min.

### Preparation of artemisone containing aqueous dispersions of PCL-b-MPEG

The dispersions were prepared according to a previously published procedure [26]. In short: solvent displacement (2.5% w/w PCL-*b*-MPEG; 2–2.5% w/w artemisione) took place. One gram of the PCL-*b*-MPEG, with different block length (a-d), and 20 mg artemisone were dissolved in 26 ml THF and poured into 39 g water. To remove the THF, the solution was stirred under a mild air stream at 20 °C for 2 days. Dispersions (a-d) were formulated in the following composition and were sterilized by filtration through 0.22 μm filters. The dispersions contained nanoparticles of about 100 nm.

- PCL_15,000_-b-MPEG_5,000_, 2.5% Polymer, 2% artemisone
- PCL_5,000_-b-MPEG_5,000_, 2.5% Polymer, 2.5% artemisone
- PCL_25,000_-b-MPEG_5,000_, 2.5% Polymer, 2% artemisone
- PCL_15,000_-b-MPEG_2,000_, 2.5% Polymer, 2% artemisone

The difference among the compounds is the hydrophilic-lipophilic-balance, which describe the hydrophobic and hydrophilic share of the polymer blocks in the block copolymer. MPEG is hydrophilic, biocompatible segment and PCL is hydrophobic, biocompatible and biodegradable segment in the polymer. The subscript numbers show the molecular weight of the polymer block. For example, sample a (PCL_15,000_-b-MPEG_5,000_) has a total molecular weight of 20,000 Da, at which the PCL has a molecular weight of 15,000 Da and MPEG has a molecular weight of 5000 Da. By comparing these two molecular weights one can estimate the degree to which a polymer is hydrophilic or lipophilic and while doing so the particle size of the block copolymer. A higher molecular weight of the hydrophobic PCL or a lower molecular weight of the MPEG leads to larger particles. The particle size is important, especially for surface degradation and consequently for the release of the drug. The smaller particles should have a higher releasing rate than the larger ones due to their higher surface-to-volume-ratio.

The PCL-b-MPEG dispersions were not toxic to THP-1 cells in vitro (Bubel, personal communication). Other dispersions were prepared with higher concentration of artemisone (5% w/w and 10%w/w) but the drug was sedimented after removal of the organic solvent.

### Macroscopic and histopathological evaluation

Surviving animals were sacrificed one and a half months after treatment with polymers containing 2 mg artemisone (80 mg/kg), and tissues from areas close to the insertion place were fixed in 4% formaldehyde solution. The tissues were processed into paraffin and 3 μm sections were stained with hematoxylin and eosin for histological evaluation. The examination parameters included necrosis and inflammatory cell infiltration.

### Bioassay for in vitro release of artemisone from PCL-b-MPEG

Released artemisone was quantified in a bioassay based on two-day cultures of the artemisone sensitive *P. falciparum* that stably expresses a luciferase gene (see parasites section above). PCL-MPEG samples were sterilized by UV exposure and transferred to 1 mL RPMI 1640 medium in 24 well, Nunc disposable sterile plates that were incubated at 37 °C. Once a day the medium was collected and frozen until use; then, the polymers were washed twice in 2 ml medium, 1 ml fresh medium was added and the plates were returned to the incubator. The collected supernatants in different dilutions were examined for *P. falciparum* growth inhibition in Nunc flat bottom 96-well plates (Nunc™ MicroWell™ 96-Well Optical-Bottom Plates with Polymer Base; Nalge, Rochester, USA). Luciferase activity was measured in parasitized erythrocytes after removing 100 μl of the medium, following addition of 100 μl Bright-GloH luciferase reagent (Promega, Madison, USA) in a Fluoroskan FL luminometer (Thermo, Paisley, UK).

### Statistics

Experiments of the present study were performed at least twice (with reproducible results) except the examination of the dispersions that was performed once because it yielded non-satisfactory results in comparison with the solid polymers. Statistical analysis was performed using GraphPad Prism, version 6.0.7 for Windows (GraphPad Software, La Jolla, CA).

Development of parasitemia was compared using Kruskal-Wallis test; delay (or prevention of death) was compared using Log RANK analysis; linearity correlation of parasitemia counted by microscopic observation and that measured by luminescence was tested using Pearson’s correlation; Student’s *t*-test was performed to compare inhibition of parasite development.

## Results

### Aqueous PCL-MPEG dispersions

**Fig. 1:**
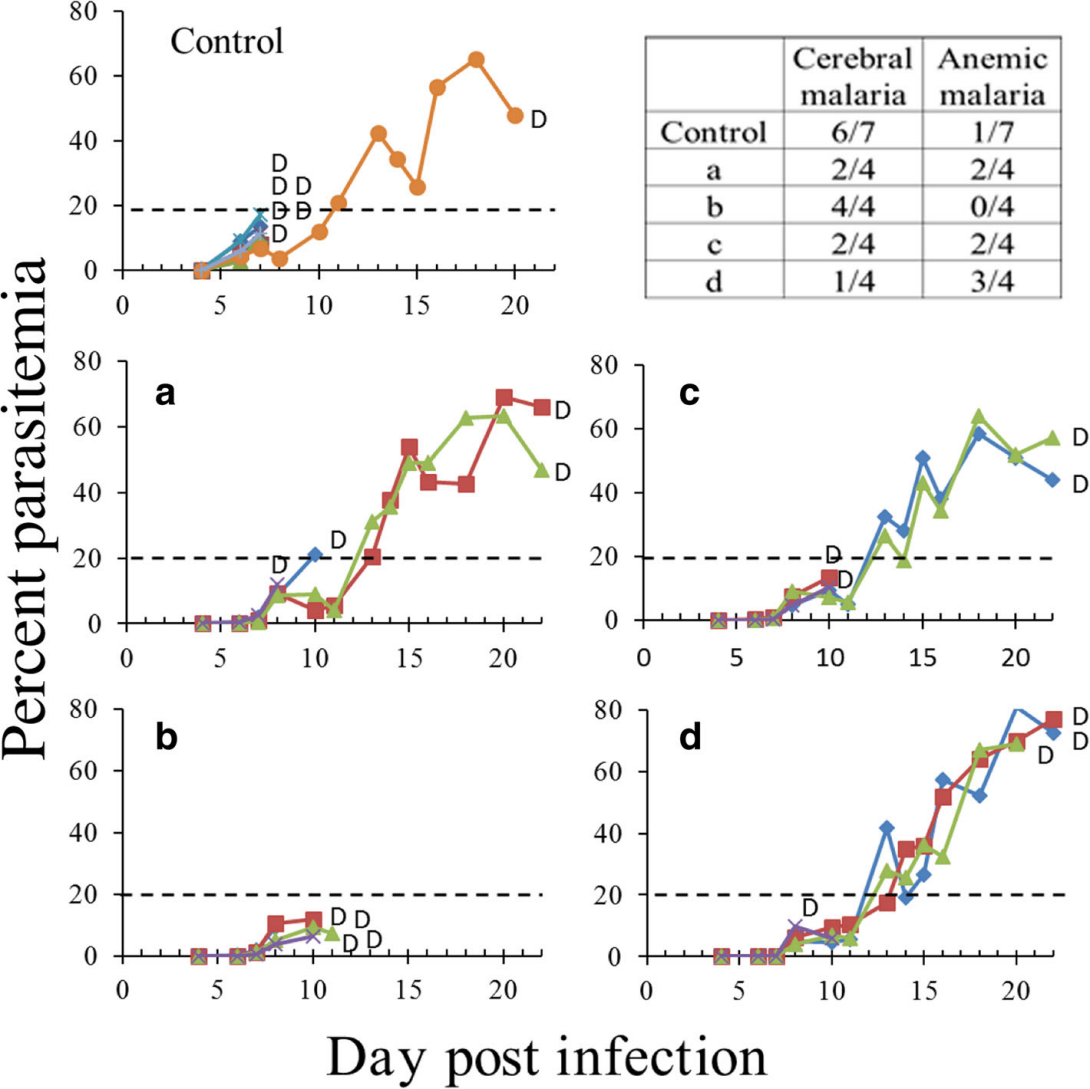
The effect of early treatment using artemisone in dispersions. **a**-**d** Different artemisone-containing dispersions (see Methods) at 6 mg/kg/injection. The dispersions were intraperitoneally injected twice a day, one and three days post-parasite inoculation. Some of the dispersions prevented CM and prolonged the survival time by about two weeks. These mice died of AM. We did not continue using dispersions because it was impossible to increase the amount of artemisone in them and because other artemisone formulations that were injected at the early stages of the disease completely eliminated the parasites (see Fig. 2)

**Fig. 2:**
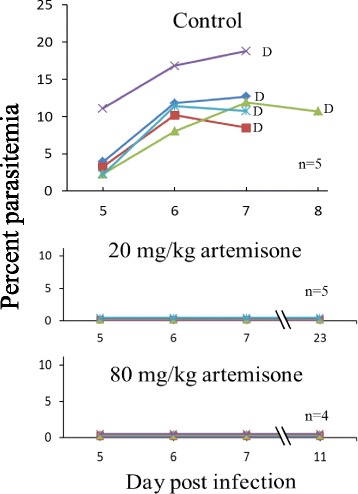
The effect of early treatment using artemisone in solid polymers. Polymers were subcutaneously inserted one day pi. Each line represents one mouse. While all control mice died of CM, the treatment significantly eliminated the parasites and all mice recovered. Identical results were obtained after insertion of the polymers two days pi (data not shown)

Repeated IP injections of various artemisone-containing aqueous PCL-*b*-MPEG dispersions revealed an effect of shifting from CM death to anemic malaria (terminated by death that occurred three weeks post-infection). However, there was no complete elimination to non-detectable parasitemia following the treatment. Despite the significant results (especially in d), the outcome was disappointing considering the early stage of the treatment (Fig. 1). We did not continue using dispersions because it was impossible to increase the amount of artemisone in them and because the other artemisone formulations that were injected at the early stages of the disease eliminated the parasites (see Fig. 2).

### Solid samples of PCL-b-MPEG

Artemisone-containing solid polymers (0.5 mm thick, 5 × 20 mm; 0.5 or 2 mg artemisone in 50 mg polymers; 20 mg or 80 mg/kg, respectively) or blank polymers were IP inserted on different days before or post-inoculation (pi) with PbA.

**Fig. 3:**
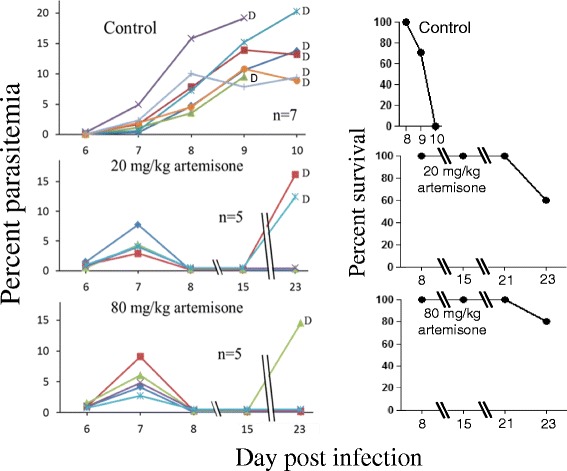
The effect of late treatment, 6 days pi, using artemisone in solid polymers. Polymers were subcutaneously inserted six days pi. Each line represents one mouse. All control mice died of CM within 10 days pi. Artemisone treatment eliminated the parasites to an undetectable level and a latent period in all mice. 3/5 and 4/5 survived after 20 and 80 mg/kg treatment, respectively. These differences were significant

**Fig. 4:**
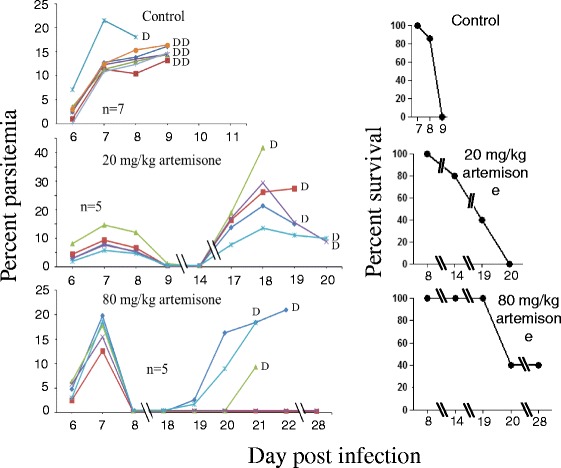
The effect of late treatment, 7 days pi, using artemisone in solid polymers. Polymers were subcutaneously inserted seven days pi. Each line represents one mouse. All control mice died of CM within 9 days pi. Delaying the treatment by one day, from 6 (Fig. 3) to 7 days pi, reduced the following latent period; mice treated with 20 mg/kg did not survive and only 2/5 mice treated with 80 mg/kg artemisone survived the infection. However, the treatment had a significant effect in reducing parasitemia to undetectable level during a latent period, and delaying mortality

**Fig. 5:**
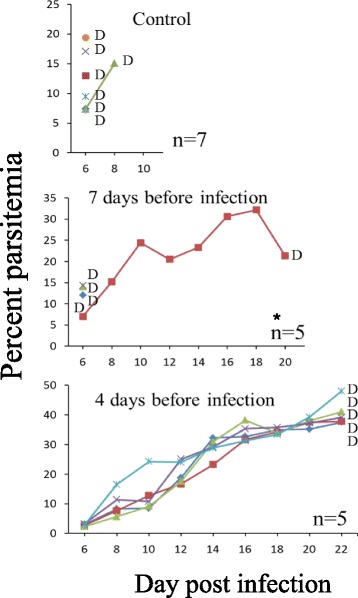
The effect of prophylactic treatment using 80 mg/kg artemisone in solid polymers. Polymers were subcutaneously inserted four or seven days before infection. Each line represents one mouse. Prophylactic treatment seven days before infection had no effect on parasitemia or survival. Treatment four days before infection prevented CM in all mice but they went on to die with AM two weeks after the death of the control mice

Throughout the experiments all control untreated (inserted with blank polymers) infected mice died of CM on days 7–10 pi (in individual experiments within two days). Mice that were treated with solid polymers containing 20 and 80 mg/kg artemisone were completely cured, if the polymers were inserted 1–2 days pi (Fig. 2). Insertion of the polymers 6 days post-infection abolished detectable parasitemia, as early as one day after the insertion. A resulting latent stage that lasted a week in all treated mice was followed by a relapse in 2/5 of the mice treated with 20 mg/kg and 1/5 in the mice treated with 80 mg/kg. The non-relapsing mice completely recovered (Fig. 3). Treatment on day 7 pi delayed death but was less efficient than the earlier treatment: after five days of latency the disease relapsed in all mice treated with 20 mg/kg artemisone. 3/5 of these mice died of anemic malaria and 2/5 died of CM. 3/5 mice treated with 80 mg/kg drug died of CM and 2/5 were completely cured (Fig. 4). Polymers containing 80 mg/kg artemisone inserted 7 days before parasite inoculation were not prophylactic: 4/5 mice died of typical early CM and 1/5 of anemic malaria 20 days pi. The results were improved with insertion of identical polymers 4 days before infection. This treatment did not prevent death from malaria; however, the treatment changed the course of the disease and instead of dying of CM all 5/5 mice succumbed to anemic malaria, 22 days pi, about 2 weeks after the death of the control group (Fig. 5).

One and a half months after insertion, the polymers were not visible. Histology did not reveal any damage to nearby tissues (data are not shown).

### In vitro release of artemisone from solid polymers

**Fig. 6:**
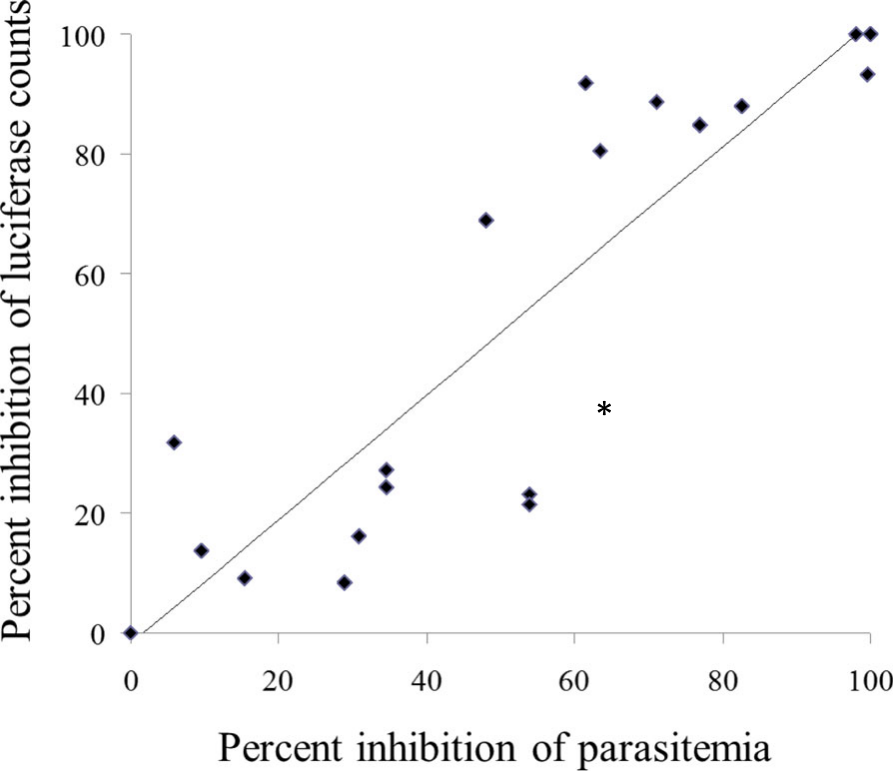
Correlation between inhibition of in vitro development of *Plasmodium falciparum* as measured by the luminometer versus microscopic observation. Various concentrations of artemisone were added to the cultures. The linear regression between the luminescence detection and the microscopic observation indicates very high correlation (*R* ^2^ = 0.78, *P* < 0.0001)

The artemisone content of supernatants collected on various days following in vitro release of the drug were estimated by both luciferase assays and microscopic observation of stained blood smears, of the same cultures. The results indicate an absolute correlation between the two methods (Fig. 6). However due to its accuracy and ease of performance, the luciferase assay was selected for the *P. falciparum* quantitation.

**Fig. 7:**
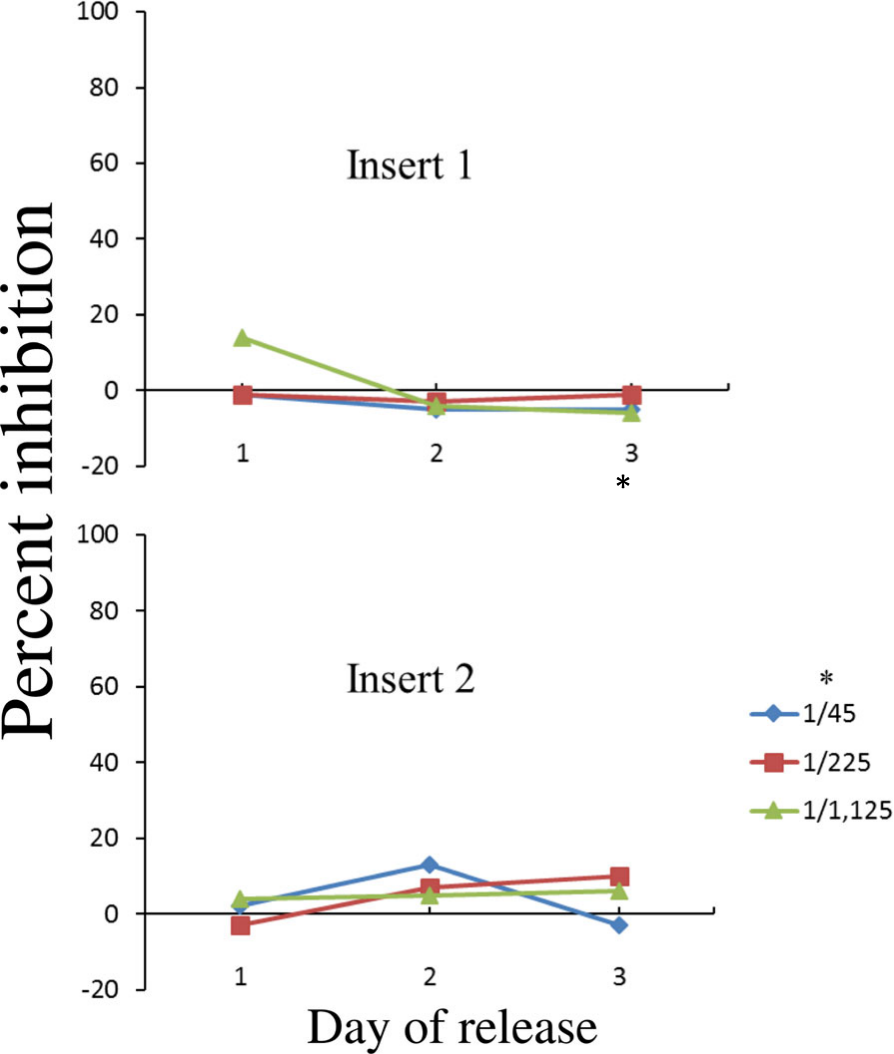
Inhibition of in vitro development of *Plasmodium falciparum* by supernatants released from media incubated with blank solid polymers. Samples were collected during three days from media incubated in vitro with blank polymers (inserts 1 and 2) and tested for their ability to kill *P. falciparum* in culture. *Supernatant dilution. The initial parasitemia was 1% and final parasitemia was 2.2% at the end of the experiment two days later. Fluorescence reading for the control culture was 22,000 Relative Luminescence Units (RLU)

**Fig. 8:**
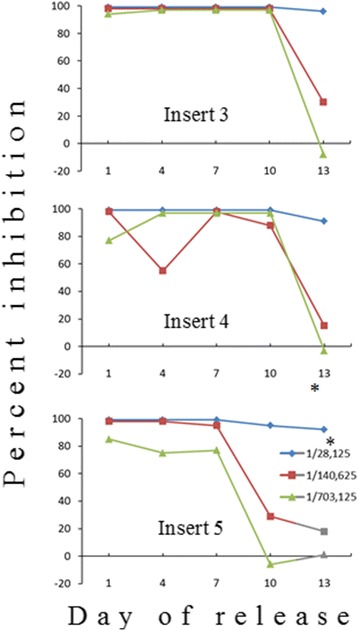
Inhibition of in vitro development of *Plasmodium falciparum* by supernatants released from media incubated with artemisone solid polymers containing 2 mg artemisone. Samples were collected during 13 days from media incubated with solid polymers containing two mg artemisone (inserts 3–5). *Supernatant dilution. The initial parasitemia was 1.5% and final parasitaemia 8.5% at the end of the experiment two days later. Fluorescence reading for the control culture was 40,500 relative luminescence units (RLU). Solid polymers containing artemisone released significant amounts of artemisone, at least until day 10 in culture

The amount of released artemisone was further estimated in cultures of *P. falciparum* that expresses the luciferase gene. Free artemisone standards were added for comparison. Supernatants from media incubated with blank solid polymers had no effect on *P. falciparum* development (Fig. 7). In contrast, considerable amounts of artemisone were released in vitro, spanning at least 13 days (Fig. 8). For example, a dilution of 1/140,000 of supernatant collected on day 7 killed most of the parasites (meaning that the amount of released artemisone was above 140 μg on that day). The ED50 of free artemisone was estimated by identical methods and was about 1 ng/ml (concentrations of 0.1–10 ng/ml were estimated in triplicates).

## Discussion

Cerebral malaria is a major cause of malarial death and its treatment is complicated because of the detrimental role of the parasite and the deleterious immunopathological response. In view of the etiology of CM, both anti-plasmodial and anti-inflammatory treatments are necessary, in addition to adjunctive therapy [27, 28]. Currently, artemisinin derivatives are used as a first line anti-malarial treatment [29]. Artemisone, being anti-plasmodial and anti-inflammatory [15], was selected for the current investigation. It is a recently synthesized artemisinin derivative with longer in vivo half-life relative to other derivatives [30], and has a superior anti-plasmodial activity [10]. However, in mouse models repeated injections, twice a day for at least three days, are necessary for a significant anti-plasmodial effect [14]. In humans, a seven-days treatment against *P. falciparum* is necessary to eliminate the parasites (and prevent CM, providing that the parasites are not resistant to the drug) [31]. Artemisinin derivatives are toxic at high concentrations that cannot be accurately controlled by the usual route of injections [13, 32]. Oral treatment may reduce toxicity. However, due to very low absorption (and other factors such as first pass metabolism), much higher doses should be applied (limiting the use of the drugs for economic reasons, unpublished data).

Although artemisone has improved pharmacokinetics compared to other artemisinins, low patient compliance may limit its use. As an alternative approach, we incorporated the drug into different biodegradable preparations, to enable its slow release. The preparations were inserted into mice as prophylactic therapy (for proving the in vivo slow release of the drug) or as an early or late treatment of experimental CM.

Previously, a limited number of experiments were performed with the aim of obtaining improved activity of artemisinins. Unfortunately, many of these experiments were performed using artemisinin, which is no longer in use in clinical treatment of malaria – only derivatives are used. Some of these earlier experiments were aimed at malaria treatment and some at cancer treatment. The solubilization capacity of micelles of sodium dodecyl sulphate (SDS) increased artemisinin solubility by 25-fold [33]. Drug solubility studies of solid dispersions of the poorly soluble artemisinin were developed using polymer blends of polyvinylpyrrolidone (PVP) and polyethylene glycol (PEG) with the aim of enhancing the drug’s solubility and skin permeation in a model system. The solubility and the permeation were enhanced, suggesting a new route for malaria treatment [34]. Polyelectrolytes were deposited on artemisinin crystals but the release of the artemisinin was measured only in vitro. The half release time from the nanoparticles was about 10 h [35]. Artemisinin delivery and treatment could be improved by using mixed non-ionic surfactants. The results of size, zeta potential and polydispersity index of niosomal formulation indicated that the size of the resulting vesicles was below 200 nm, their surface charge about -35 mV and they were monodisperse. The PEGylated formulation had a stable release pattern and a greater anti-tumor activity on cancer cell lines than the free drug [36]. Ibrahim et al. report [37] the preparation, characterization, and in vitro and in vivo biological evaluation of biodegradable albumin-bound artemisinin nanoparticles. The nanoparticles were prepared by a combination of a bottom-up and a top-down processes and were suitable for intravenous injection. The results indicate improved anti-plasmodial activity of the nanoparticles over artemisinins in *P. falciparum* cultures and in humanized severe combined immunodeficiency (SCID) mice.

Another artemisinin derivative, artether, loaded in lipid nanoparticles had improved bioavailability in rats. However, the artether’s half-life was only approximately three hours [37]. Artesunate (the most used artemisinin derivative) loaded in chitosan/lecithin nanoparticles was adapted for controlled release. Oral treatment induced protection in infected mice for about one month. However, to obtain the effect, the mice were treated twice a day for seven days (14 interventions), starting one day post inoculation of the plasmodia (when minimal amounts of parasites are present) [38]. Dwivedi and colleagues described the entrapment of artemisone in solid lipid nano-particles and niosomes, and demonstrated that the entrapment improved the efficacy of artemisone against a melanoma cell line with negligible in vitro toxicity towards human keratinocytes [39].

Using a reliable mouse model of CM, throughout our experiments all mice that did not receive drug treatment died of CM. We used for treatment biodegradable formulations, either artemisone-containing dispersions or solid polymers. Dispersions at 6 mg/kg/injection were intraperitoneally injected twice a day, one and three days pi, to infected mice. Some of the dispersions prevented CM and prolonged the survival of the treated mice by about two weeks. The mice with extended survival died later of AM. The small (50–100 nm) dispersions have the advantages of easy sterilization by filtration and simple administration by injection; however, we did not continue using them because it was impossible to increase the amount of artemisone in the dispersions, repeated injections were a must, and other formulations that were inserted at the early stages of the disease eliminated the parasites. Solid polymers could easily be sterilized by UV exposure. Unlike the dispersions, the solid polymers cured all mice treated by a single insertion during the first few days pi. Later in the course of infection, when symptoms of CM were obvious, it was still possible to change the course of the disease by treatment with the solid polymers: artemisone-containing polymers, 20 and 80 mg/kg, cured most of the mice (60 and 80% cure, respectively) when used six days pi. Even later, at seven days pi (all control mice would die of CM within 24 h), insertion of 80 mg/kg artemisone in polymers saved 40% of the mice. Most importantly, in all cases of late treatment parasites were not detected for about a week. In those mice that were not completely cured a relapse followed the latent period. This delay is vital because, if replicated in humans, it would enable at least an extra week for exact diagnosis and appropriate treatment. Misdiagnosis in malaria-infected persons and the consequent lack of appropriate treatment often leads to death or long-term cognitive defects [40]. Intraperitoneal injection of artemisone had toxic effects (80 mg/kg in 40 μl DMSO killed 25% of the mice, DMSO had no effect, data not shown).

We also examined the solid polymers in prophylactic experiments. Following polymer insertion that had been carried out seven days before infection, the released artemisone had no effect on the course of the disease; in contrast, CM prevention was demonstrated after polymer insertion four days prior to the infection. These results enable an estimation of the amount of artemisone that is released from the PCL-*b*-MPEG since it was reduced to an ineffective level after four days in vivo. In parallel, we measured the amount of artemisone that was released from the rigid polymers using an in vitro *P. falciparum* bioassay. In this assay, we measured the luminescence of transgenic parasites, an approach that was in absolute correlation with microscopic observation for parasite estimation. According to the reference artemisone quantification, at least 1 microgram artemisone/day was released from the polymers until day 10 of the bioassay. Later, there was a decline in release down to non-significant quantities by day 13. Overall, there was a parallel between the in vitro bioassay and the in vivo results that shows the suitability of the bioassay for predicting the amount of artemisone that would be released in vivo. The amount of drug that we used would be toxic if injected as a single dose [13, 32], illustrating another advantage of the controlled release strategy.

## Conclusions

Solid polymers that contain artemisone were inserted subcutaneously and released the drug during at least a week in non-toxic quantities that were sufficient to prevent or delay CM in a mouse model, even when applied at a very late stage of the disease. We demonstrate as a proof-of-concept this controlled-sustained release system for safe and effective treatment of malaria, emphasizing the advantage of treatment of CM where conventional mode of treatment is complicated. Similar methods could be used for other parasites that are sensitive to artemisinins.

## Abbreviations

AM: Anemic malaria
ACTs: Artemisinin combination therapies
CM: Cerebral malaria
DHA: Dihydroxyartemisinin
IP: Intraperitoneally
PbA: *Plasmodium berghei* ANKA
PEG: Polyethylene glycol
pi: Post-inoculation
PVP: Polyvinylpyrrolidone
SCID: Severe combined immunodeficiency
SDS: Sodium dodecyl sulphate
THF: Tetrahydrofuran
